# Stimulatory Effect of Morning Bright Light on Reproductive Hormones and Ovulation: Results of a Controlled Crossover Trial

**DOI:** 10.1371/journal.pctr.0020007

**Published:** 2007-02-09

**Authors:** Konstantin V Danilenko, Elena A Samoilova

**Affiliations:** 1 Centre for Chronobiology, Institute of Internal Medicine, Siberian Branch of the Russian Academy of Medical Sciences, Novosibirsk, Russia; 2 Medical Centres Tet-a-tet and Phytomed, Novosibirsk, Russia

## Abstract

**Objectives:**

Studies have shown a shortening of the menstrual cycle following light exposure in women with abnormally long menstrual cycles or with winter depression, suggesting that artificial light can influence reproductive hormones and ovulation. The study was designed to investigate this possibility.

**Design:**

Placebo-controlled, crossover, counterbalanced order.

**Setting:**

Medical centres and participants' homes in Novosibirsk (55°N), Russia.

**Participants:**

Twenty-two women, aged 19–37 years, with baseline menstrual cycle length 28.1–37.8 d and no clinically evident endocrine abnormalities completed the study. The study lasted for two menstrual cycles separated by at least one off-protocol cycle.

**Interventions:**

During one experimental cycle, bright light was administered at home for 1 wk with a light box emitting white light at 4,300 lux at 41 cm for 45 min shortly after awakening. During the other experimental cycle, dim light was <100 lux at 41 cm with a one-tube fluorescent source.

**Outcome Measures:**

Blood samples and ultrasound scans were obtained in the afternoon before and after the week of light exposure, on day ∼7 and 14 after menstruation onset. Further ultrasound scans after day 14 documented ovulation. Serum was assayed for thyroid-stimulating hormone (TSH), prolactin (PRL), luteinizing hormone (LH), follicle-stimulating hormone (FSH), and estradiol (E2).

**Results:**

Concentrations of PRL, LH, and FSH were significantly increased with bright versus dim light exposure, as was follicle size (ANOVA, intervention × day, *p* = 0.0043, 0.014, 0.049, and 0.042, respectively). The number of ovulatory cycles increased after exposure to bright compared to dim light (12 versus 6 cycles, Wilcoxon tied *p* = 0.034).

**Conclusions:**

Morning exposure to bright light in the follicular phase of the menstrual cycle stimulates the secretion of hypophyseal reproductive hormones, promotes ovary follicle growth, and increases ovulation rates in women with slightly lengthened menstrual cycles. This might be a promising method to overcome infertility.

## Editorial Commentary

**Background:** It is not clear whether light plays any role in determining menstrual cycles or ovulation in women. However, light is used as a treatment for some medical conditions related to rhythm, most notably seasonal affective disorder. In this study, the researchers wanted to examine the role of light in influencing the menstrual cycle. In the trial reported here, 22 women with lengthened menstrual cycles were provided with a light box that emitted either bright light or dim light, and the women were asked to use the light box for a defined period each day for one menstrual cycle. The study had a crossover design, where women would receive either bright or dim light for one cycle, then another cycle without the light box, and finally a cycle with either bright or dim light, whichever they did not receive first. Outcomes assessed in the trial included measurements of the blood levels of certain hormones. Specifically, the researchers looked at three hormones that determine the reproductive cycle (lutenizing hormone [LH], follicle stimulating hormone [FSH], and prolactin); these are produced by a region of the brain that is controlled by the hypothalamus, a gland that is directly responsive to light. Additionally, ultrasound scans were used to check for ovulation during each cycle. The trial was carried out in winter in Novosibirsk, a Russian city.

**What the trial shows:** When the researchers compared hormone levels between the “bright light” and “dim light” phases of the study, they found statistically significant increases in levels of LH, FSH, and prolactin in the blood. Ovulation was more likely in the “bright light” phase of the study as compared to the “dim light” phase. However, levels of two other hormones, thyroid stimulating hormone, and estradiol, were not significantly different when comparing the “bright” and “dim” cycles.

**Strengths and limitations:** In this trial the use of a crossover design enabled each woman to act as her own control, thereby reducing the number of participants that were needed in the trial. A further strength in this study was the use of ultrasound scans to allow the researchers to pinpoint ovulation in the participants; similar studies have not tried to examine the effects of light both on reproductive hormones and ovulation. A weakness of the design is that participants were assigned to receive either dim light first or bright light first using alternation, not true randomization. Therefore blinding was not possible and the researchers recruiting participants would have known in advance what intervention each participant would receive first. Other weaknesses include the limited number of participants in this study, and the nature of the participant population, all of whom had lengthened menstrual cycles and were living at fairly northerly latitude.

**Contribution to the evidence:** This study adds data suggesting that in women with lengthened menstrual cycles, ovulation can be stimulated by bright light. However, this finding would need to be replicated in a larger sample of women and it is not yet clear whether bright light will have the same effect on ovulation in women outside northerly latitudes and with average-length menstrual cycles.

## INTRODUCTION

Artificial (ocular) light administered at the appropriate time of day is used to overcome a variety of conditions associated with circadian rhythm abnormalities (shift work, jet lag, sleep disturbances), monthly cycles (premenstrual dysphoric disorder), seasonal variations (winter depression, bulimia nervosa), amongst others [1]. Forty years ago it was asserted that light might regulate the menstrual cycle and ovulation [2]. A series of six studies—one in the Boston area, four in San Diego, and one in Novosibirsk (1965–1995)—have shown that the menstrual cycle shortened in women with long menstrual cycles after bedside light administered overnight around the days of presumed ovulation [3–7]. Two subsequent studies found an increased rate of ovulation determined with a home dip-stick test detecting an ovulatory surge of LH in urine ([8], Putilov et al., unpublished data]. Additional evidence came from a group of patients with seasonal affective disorder who received conventional morning bright light therapy or dawn simulation for a week starting within 0–13 days after menstruation onset. This resulted in a shorter menstrual cycle by 1.2 days on average, and the effect was not correlated with the depression improvement [9].

The above studies were not accompanied by the hormonal measurements that might elicit the mechanisms underlying the cycle-shortening effect of light. Surprisingly, there has been little investigation in humans into the effects of light on the two principal hormones regulating the menstrual cycle—follicle-stimulating hormone (FSH) and luteinizing hormone (LH)—compared with other hypophyseal hormones and melatonin. These studies showed an increase of LH and/or FSH, in men or women, following bright light 500 to 3,000 lux [10–12].

The aim of this study was to answer conclusively whether light can influence menstrual cycle and ovulation, and to determine the underlying hormonal changes taking place. The design combined several approaches used in previous studies and advanced some of them. For example, ultrasound scans were introduced as a method to objectively document ovulation. Bright light, during wakefulness was used since it has also been shown to shorten the menstrual cycle [9], does not disturb sleep (as dim overnight lights might) and is better studied (in terms of neuroendocrine effects). The dark season and the hour immediately following wakening were chosen as the exposure time since light stimulus is most effective when contrasted with low levels of light in the preceding period (e.g., [13]). Women with slightly lengthened menstrual cycles were selected with a view to shortening the cycle, i.e., a sort of treatment since in woman with normal cycles this shortening could be undesirable [9]. On the other hand, women with very long menstrual cycles (mean >42 d) are only slightly responsive to light exposure [7], suggesting that significant hormonal abnormalities are not subject to a relatively subtle intervention such as light therapy.

## METHODS

### Participants

The study was performed in Novosibirsk (55°N), in three consecutive years 2003–2006, between October and April (the shortest day is 7 h). Test participants were recruited via advertisements in a local newspaper and a patient database of the gynaecologist (EAS). The advertisement was as follows: “Women with lengthened menstrual cycles (30–38 days) are invited to participate in a study. Ultrasound and hormonal investigations will be conducted free of charge”. Respondents provided dates of menstruation onset over the past year and suitable candidates were invited for clinical interview. During the interview with the investigators (both are clinicians), they received a detailed description of the study, completed a questionnaire concerning their general health and answered some medical questions from the clinicians. The inclusion criteria required that the majority of menstrual cycles, especially the last two baseline cycles, were free from any medication known to interfere with hormone release; the absence of clinically evident endocrine abnormalities; good general health; a normal sleep-wake regimen; and no transmeridian travel two months prior to the study.

After meeting the inclusion criteria, the candidates signed the consent form if they were willing to participate. The most common motive for participation was long-standing difficulty in conceiving and/or a physician's recommendation for a hormonal investigation, which is expensive. The consent included the statement: “The agreement provides the participant with free testing and is offered on a mutually beneficial basis: the participants receive information regarding their reproductive system, whilst the clinicians are able to investigate the effects of light”. The study was approved by the Ethics Committee of the Institute of Internal Medicine SB RAMS.

### Protocol

The study lasted for two menstrual cycles separated by an off-protocol episode of at least one menstrual cycle. Bright light was administered during one experimental cycle, dim light during the other (placebo-controlled, crossover, counterbalanced order design). Light treatment lasted for a week at home and was preceded by the day of baseline investigations and followed by the day of follow-up investigation. Both the baseline and follow-up investigations were planned to be performed in follicular phase before ovulation, because the dominant follicle ruptures and the hormonal profile changes very considerably on ovulation and this might confound consistency of our results, especially if not all cycles are ovulatory. On the other hand, light exposure is most effective when administered around the days of presumed ovulation [7]. Therefore, the approximate day for entering the study was calculated as minimal cycle length (for a particular woman) minus 14 d (usual duration of the luteal phase) and minus 7 d (duration of the light treatment). Participants came to a medical centre in the afternoon to give 5 ml of 3-h fasted venous blood and to pass an ultrasound examination. They took a light device and a diary back home to start daily light exposure the next morning, and returned a week later (on the day of the last light exposure) for the follow-up examination. For simplicity, baseline and follow-up examination days are referred to in the text as “day 7” and “day 14”. Ultrasound scans were conducted as required to document ovulation and participants also recorded post-awakening rectal temperature daily in their diary to pinpoint the date.

### Interventions

**Figure 1:**
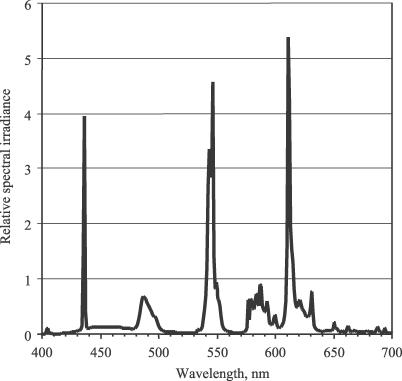
Spectral Composition of the Bright Light The figure shows a spectral power distribution of the light emitted from a Sunray2-Max box (Outside In). It uses phosphor type 835 lamps (data from Sylvania Lighting). The short wavelength up to ∼450 nm was significantly attenuated by the diffuser (filter characteristic from Interlux).

As in study [9], Sunray2-Max box (Outside In) was used to provide bright white light of intensity 4,300 lux. The lamps have a phosphor type 835 with a correlated colour temperature of 3,500 K (Figure 1). The participant was instructed to sit at a distance of 41 cm facing the screen of the box for 45 min shortly after waking. It was not necessary to look at the screen all the time, just to allow light to freely enter both eyes. Dim light (placebo control) was of normal room intensity less than 100 lux at 41 cm from a domestic 1-tube fluorescent fitting. After each week of light intervention, the participant was invited to comment on their experience of using light. No specific monitoring of adverse events was done (e.g. eye examination) because the light is known to be a low-risk intervention [1].

### Objectives

The objective of the study was to investigate the influence of morning bright light on ovarian function in women.

### Outcomes

Blood was drawn from the antecubital vein, centrifuged within 1 h, and the serum was kept frozen until the hormonal assay. Serum was assayed for concentrations of thyroid-stimulating hormone (TSH), prolactin (PRL), LH, FSH by immunoluminometric assay (ILMA), and for estradiol (E2) by ELISA. Reagent ILMA kits were obtained from Immunotech (http://www.beckmancoulter.com/products/pr_immunology.asp), and ELISA kits from DRG (http://www.drg-diagnostics.de). The samples from each individual were assayed in the same run, using one kit, to avoid interassay variability. The sensitivity of the assay and maximal intra-assay variations within the normal range of hormone were: 0.03 mU/l and 6.2% for TSH, 0.26 ng/ml and 3.4% for PRL, 0.2 U/l and 5.6% for LH, 0.2 U/l and 2.9% for FSH, 9.7 pg/ml and 6.8% for E2. Based on the literature, TSH and PRL are secreted in a rhythmic fashion with an increase at night-time and relatively stable values during an afternoon trough, whereas LH, FSH, and E2 have no distinct 24-h variations (e.g., [14]). Blood was taken in the afternoon in order to exclude any confounding effect on the results of any phase shift of the hormone's circadian rhythm by repeated light stimuli. Since LH shows pulsatile secretion during the day (e.g., [15]), a half-day urine sample was collected in a subset of participants, to be analysed in the event of near-significant results on serum LH.

Ultrasound examination was performed using a linear/sector ultrasonic compound scanner SSD-500 (Aloka [http://www.aloka.com/]) with 3.5-MHz transabdominal and 5-MHz transvaginal convex probes. Follicle diameter, ovary volume, and endometrium thickness were estimated and the scan images/results printed. Follicle growth was determined as the difference in diameter of the maximal (dominant) follicle on day 14 and maximal follicle in the same ovary on day 7. The disappearance of a dominant follicle in subsequent scans indicated that ovulation had occurred. The presence of free fluid behind the uteri helped to show ovulation had occurred recently, and an increase in echo signal from the endometrium confirmed the transition from preovulatory to postovulatory phase.

### Sample Size

No formal sample size calculation was done. In the previous light exposure studies, 8 to 27 participants were analysed regarding menstrual cycle length [3–8], 27 and 13 regarding ovulation ([8], Putilov et al., unpublished data), and 11 to 17 subjects regarding the hormones of main interest LH and FSH [10–12]. With our crossover design, we aimed to have at least 15 participants complete the study, targeting 20–25 to either obtain a definitive conclusion within two years, or possibly to halt the study after one year if interim analysis of the results showed no significance. In fact, the study period was extended to three years because of operational problems.

### Randomization

Entering the study was determined by participants' menstrual cycle onset and, therefore, was sequential and not dependent on investigators. The type of light intervention was allocated to participants alternately: the first received dim light; the next, bright light; the third, dim; etc. Alternating in this way helped to better balance between months (seasons) and also utilized fewer light devices.

### Blinding

The study participants were not told whether they were to received dim or bright light first and would have realised only during the second experimental cycle. Additionally, an interest in the study's diagnostic tests rather than the intervention helped to lower expectations regarding placebo and active light effects. One of the investigators (EAS) was blind to the intervention type.

### Statistical Methods

Analysis of variance (ANOVA) with two repeated measures, intervention (bright versus dim light) and day (day 7 versus day 14), were the primary statistics in the study. Standard deviations of the means are reported in the text and tables while standard errors of the mean are in figures. Categorical or nominal variables (e.g., occurrence of ovulation) were analysed using nonparametric statistics.

## RESULTS

### Recruitment and Participant Flow

**Figure 2:**
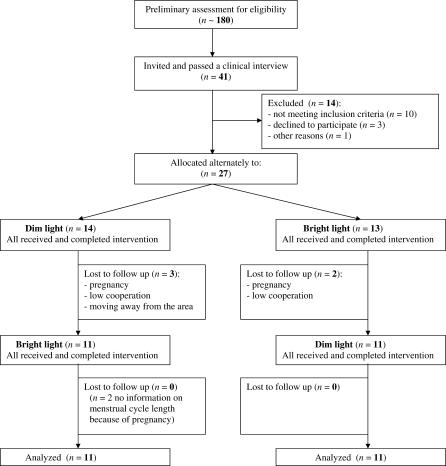
Participant Flow Chart

Participant flow is shown in Figure 2. Recruitment took place in September through November, 2003–2005. In the first year, six participants were recruited (five have ended); in the second, 8 (5); in the third, 13 (12). Of the 27 women that entered the study, five failed to complete it due to pregnancy (*n* = 2), low cooperation (*n* = 2), and moving away from the area (*n* = 1).

### Baseline Data and Numbers Analyzed

The final study group of 22 were aged 19 to 37 years (Table 1). Based on diaries of the participants, the mean menstrual cycle length ranged from 28.1 to 37.8 d, minimal cycle 26 to 32 days (median = 28 d), maximal cycle 30 to 56 days (median = 38 days), 2–12 cycles analysed (median = 8 cycles).

**Table 1:**
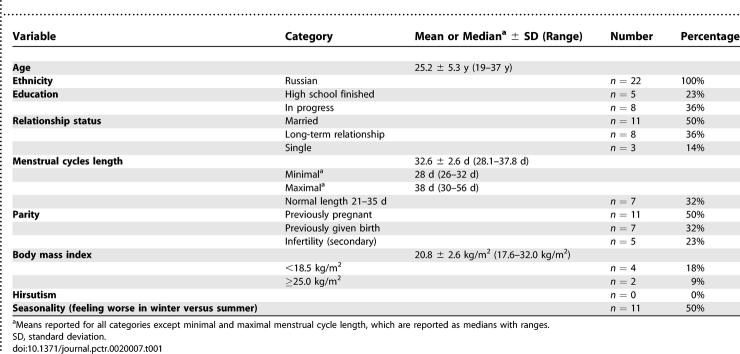
Baseline Characteristics of Women Completing the Study (*n* = 22)

Actually, the participants entered the study on day 5–9 after menstruation onset (median = 7 d, 1-d maximal difference between the two experimental cycles, except in two cases of 2 d). Light exposure started at 07:43 ± 0:55 for the dim light session versus 07:59 ± 0:59 for the bright light session (*p* = 0.069), with the individual difference between two sessions less than 1 h in all but two women (median = 20 min). The time lag between awakening and starting treatment was similar: 16 ± 14 min for the dim light versus 20 ± 17 min for the bright light (*p* = 0.16). The maximal difference between two blood sampling times on day 7 and day 14 was 2 h 45 min (median = 50 min).

Deviations of hypophyseal hormones from the normal range were rare and mild in the studied group—not higher than twice the upper limit and not below the lower limit. One woman's levels of TSH were consistently above the norm, i.e., for all four measurements, indicating a subclinical hypothyroidism. Three women had increased values of PRL, one of whom also had an episodic small increase in TSH and LH. Two more women showed elevated LH or FSH during baseline measurements. The ovarian hormone E2 was typically around the lower limit of the normal range. Almost all women had multifollicle ovaries by ultrasound scan, indicating frequent anovulatory cycles [16]. None met sufficient criteria for polycystic ovary syndrome [17].

### Outcomes and Estimation

**Figure 3:**
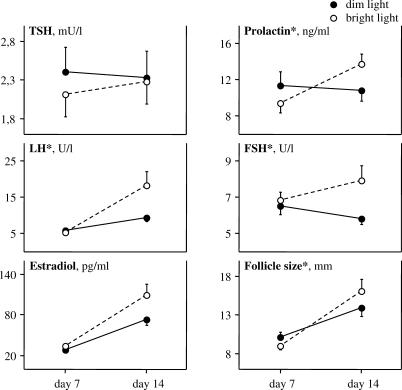
Dynamics of Hormones Secretion and Ovarian Follicle Size following a Week of Bright Light Exposure Twenty-two women with slightly lengthened menstrual cycles were exposed to bright or dim ocular light (45 min after waking, daily, for 1 wk between days ∼7 and 14 after menstruation onset, i.e., before ovulation) during two different menstrual cycles. Prolactin, luteinizing hormone (LH), follicle-stimulating hormone (FSH), and ovarian follicle size were significantly (*) increased in with bright versus dim light. The changes in thyroid-stimulating hormone (TSH) and estradiol levels were not significantly different between the two conditions (ANOVA, intervention × day interaction). Error bars indicate standard errors of the mean.

The dynamics of the hormones' concentrations, as well as ovarian follicle growth following the repeated light stimuli, are demonstrated in Figure 3; the statistics are summarized in Table 2. PRL, LH, FSH, and follicle diameter were significantly increased after bright light compared to the dim light whereas the changes in TSH, E2, and ovary volume were not significantly different between the two conditions. LH and E2 levels, and follicle and ovary sizes, were all significantly augmented after either bright or dim light (*p* < 0.01, Student's paired t-test), reflecting the normal dynamics through the follicular phase, whereas PRL concentration increased only after bright light (*p* = 0.0021). Post-treatment LH and FSH levels were within the norms for the preovulatory phase (except three cases of high LH after bright light), and PRL remained less than twice the upper limit.

**Table 2:**
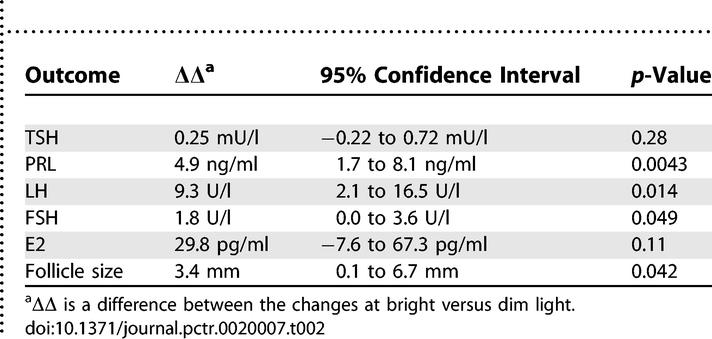
Main Outcomes and Statistical Testing for Comparisons between the Effects of Bright versus Dim Light

Separately analyzing the general ANOVA independent factors “order of treatment” (exactly half of the participants started the study with dim light first), “seasonality” (exactly half of the participants reported feeling worse in winter versus summer in the screening questionnaire), and “start month” (12 participants started the study before November) did not reveal any significant effect of these factors (either main or at interaction with repeated measures “intervention” and “day”) on the above outcomes.

The number of ovulatory cycles was higher with bright versus dim light (12 versus six cycles). Distribution was as follows: five participants ovulated during both dim and bright light cycles; nine had anovulatory cycles in both experimental conditions; one ovulated during the dim but not bright light cycle; and seven vice versa (tied *p* = 0.034, Wilcoxon test).

Menstrual cycle length was not significantly different between dim versus bright light (33.2 ± 4.8 d versus 32.7 ± 5.6 d, *n* = 20; tied *p* = 0.71, Wilcoxon test). Two menstrual cycles were missed from the analysis because of pregnancies during the second-order experimental cycle (Figure 2).

### Ancillary Analyses

In total, four women became pregnant during the study period (though all were asked to use condoms during intercourse)—three during the bright light experimental cycle and one during the dim light cycle. In the first three women, ovulation occurred at days 17–18 of the cycle, shortly after the light treatment ended. In the fourth case conception occurred late: at day 24, the dominant follicle was still not present, and the ultrasound examination was stopped. The prospective ultrasound scan 32 d later (because of absence of menstruation) and the temperature data suggested ovulation occurred on day ∼34. All pregnancies were desired.

Numerous significant intercorrelations were found between the studied hormones, follicle growth and ovary volume after light stimuli (*n* = 44), all of which were direct and many predictable. For example, changes in PRL, LH, FSH, and E2 all intercorrelated (*p* < 0.05), the strength being maximal between LH and FSH (*p* < 0.001); the only exception was no link between the changes in FSH and E2. The change in E2 most strongly correlated with the growth of the ovarian follicle (*p* < 0.001) and with ovulation occurrence (*p* = 0.006, Spearman's correlation test) which is to be expected, since most of the E2 is secreted by growing follicles.

### Adverse Events

There were no adverse effects except two reports of transient mild eye strain known to occur during bright light therapy [1].

## DISCUSSION

### Interpretation

The study showed that PRL, LH, and FSH secretions, and ovarian follicle growth and the likelihood of ovulation are all increased following a week of morning bright light exposure compared with dim light administered to the same women, in the follicular phase of their cycles. The change of TSH and E2 secretions was not significantly different between two conditions.

This is the first study to focus on the effect of light on the ovulatory process with concomitant measurement of several hormones to understand the underlying mechanism. It is clear that the increased rate of ovulation following bright light exposure is a consequence of faster follicle maturation. Follicle maturation, in turn, is determined by the complex interrelated changes in the secretion of pituitary-ovarian hormones. The neuroanatomical basis for the effect of light may include the novel melanopsin-based photoreceptors in the eye conveying information via the retinohypothalamic tract (separate from the optic nerve) to the biological clock in the suprachiasmatic nucleus of the hypothalamus (reviewed in [18]). There are likely to be numerous other links from the hypothalamus, yet the pathway to the pineal gland—secreting the darkness neurohormone melatonin—is the only one well studied. However, contrary to animal findings, there is no solid evidence that melatonin plays a significant role in the modulation of the human reproductive function (summarized in [19]). Light may act directly on the hypothalamic-pituitary-ovarian axis as it might for the hypothalamic-pituitary-adrenal axis [20]. The neuroendocrine mechanism involved in the stimulatory effect of light may be different from those regulating the monthly increase of LH and FSH during the follicular phase. To illustrate this, in Japanese quail barbiturate anaesthesia, which blocks an LH surge during ovulatory cycle, did not block the preovulatory-like increase of LH induced by light [21].

### Generalisability

The effects of light discussed here should not be generalized to women with normal or very long/irregular menstrual cycles (as these groups are likely to have different endocrinology from that of the study participants), or to the summer season and Southern locations (both providing bright rather than dark conditions).

### Overall Evidence

The literature search was done via our own database of publications on the topic, PubMed (http://www.ncbi.nlm.nih.gov/entrez/query.fcgi?DB=pubmed), and local libraries, with e-mail inquiries to the authors on specific questions, if necessary. Only four previous experiments have investigated the effect of artificial light on the reproductive hormones LH, FSH, and E2 in humans, and those results are in line with ours. In a crossover, placebo-controlled study of LH excretion, the 24-h LH level was increased after morning bright light 1,000 lux administered to 11 healthy men between 05:00 and 06:00 for 5 d [12]. Miyauchi et al. [10] investigated the effect of an acute light stimulus (3,000 lux presented between 22:40 and 24:00) on five women in their follicular phases and found an increase of LH and FSH compared to prestimulus levels 21:30–22:40. In the reference group of six participants without light, there was no change in hormone concentrations. In a subsequent study [11] the serum concentration of FSH was increased at 02:00 (*n* = 17), but not at 22:00 following light stimulus of 500–800 lux presented from 17:30 to 02:00; the change in LH did not reach a significance threshold (the authors suggested this may be because of LH pulsatility). Graham et al. [22] found no change in all-night E2 levels in 22 women exposed to bright light 5,200 lux from 21:00 to 01:00. The changes in reproductive hormones resemble a natural increased pituitary–ovarian axis activity in spring found in one [23] but not all studies performed in Finland [24,25].

TSH secretion is generally rigidly coupled to artificial light exposure (e.g., [26]) as was the case in our study. The findings on PRL in healthy participants are not consistent. In two studies, plasma PRL concentrations were increased, either during evening bright light [10] or after morning light exposure [27]. In others, PRL levels were unaffected by night light [28,29], and also after daily light exposure [30]. In some studies, PRL levels were suppressed by light at night [11,31].

The low rate of ovulation (27%) in the studied group is not surprising as menstrual cycles of over 35 days are associated with low rates of conception in a significant number of women [32]. We did not find a significant shortening of the menstrual cycle following light exposure. Whilst this was also the case in the last menstrual cycle study of the San Diego group [8], in previous studies the shortening effect had been shown consistently [3–7,9]. The cause of this discrepancy is unclear.

### Study Limitations

The limited number of participants and somewhat narrow sample population are the major limitations of the study. Another consideration is that knowledge of the study results after each of the two consecutive interim analyses may have the potential to bias the results of the following year.

### Conclusions

This study, using ultrasonographic examination and hormonal assays, shows conclusively, to our knowledge for the first time, that ovulation may be successfully potentiated by morning artificial bright light. This might be a promising method to overcome infertility in some women.

## SUPPORTING INFORMATION

CONSORT Checklist Click here for additional data file. (44 KB DOC)

Trial Protocol (Russian) Click here for additional data file. (63 KB DOC)

Trial Protocol (English) Click here for additional data file. (58 KB DOC)

## Abbreviations

E2: estradiol
FSH: follicle-stimulating hormone
LH: luteinizing hormone
PRL: prolactin
TSH: thyroid-stimulating hormone
