# Supplementary material for: Stimulatory Effect of Morning Bright Light on Reproductive Hormones and Ovulation: Results of a Controlled Crossover Trial
Source: PLoS Clin Trials. 2007 Feb 9;2(2):e7. doi: 10.1371/journal.pctr.0020007 (PMC1851732; doi:10.1371/journal.pctr.0020007)
Supplement: Trial Protocol (Russian) [file pctr.0020007.sd002.doc]

НИИ терапии СО РАМН

г. Новосибирск

"_____" _____________ 2003 г.

**ОПИСАНИЕ ИССЛЕДОВАНИЯ "M-s3"**

**Влияние искусственного света на овуляцию и менструальный цикл у человека**

**I. ОБЩИЕ СВЕДЕНИЯ**

Исследование "Влияние искусственного света на овуляцию и менструальный цикл у человека" планируется к проведению ст. науч. сотрудником НИИ терапии СО РАМН, канд. мед. наук Даниленко Константином Васильевичем (ответственный исполнитель) и врачом-гинекологом медицинского центра "Тет-а-тет" Самойловой Еленой Анатольевной в период с сентября 2003 г. по май 2005 г. К исследованию приглашаются 25-40 лиц с удлиненным менструальным циклом.

С 1967 по 2002 год в Сан-Диего и Новосибирске проведено около 8 экспериментов по влиянию искусственного света на овуляцию и менструальный цикл у женщин с удлиненным циклом (см. обзор в Putilov AA, Danilenko KV, Protopopova AY, Kripke DF. Menstrual Phase Response to Nocturnal Light. Biol Rhythm Res, 2002, 33:23-38). Начиная с 1989 года, укорочение менструального цикла систематически наблюдается у лиц с зимней депрессией в ответ на проведения курса светолечения (Даниленко, неопубл. данные). Тем не менее, УЗИ и гормональное обследование как наиболее объективные методы верификации полученных результатов не были использованы в проведенных исследованиях. В 2003 году получены предварительные данные, что свет стимулирет экскрецию лютеинизирующего гормона у мужчин (Yoon et al., 2003).

**Цель:** Оценить влияние искусственного яркого света на овуляцию и менструальный цикл у человека

**Задачи:**

(1) оценить влияние яркого света на секрецию ряда гормонов

(2) оценить влияние яркого света на фолликулогенез, овуляцию и длину менструального цикла.

**II. ОБЪЕКТ И МЕТОДЫ ИССЛЕДОВАНИЯ**

**Исследуемые**

Планируется участие 25-40 женщин с удлиненным менструальным циклом Поиск через объявления в местной прессе /**Приложение 1**/ и поток гинекологических больных в профильных медицинских учреждениях города. Отбор при личной встрече с ответственным исполнителем Даниленко  К.В. и врачом Самойловой Е.А. на основе заполнения волонтером Входной анкеты /**Приложение 2**/ и его ознакомления с исследованием, представленного в Памятке /**Приложение 3**/. Подписание Информированного согласия /**Приложение 4**/ при соблюдении всех критериев включения и исключения.

Критерии включения:

• возраст 18-40 лет

• индекс массы тела 18-30

• длина цикла за прошедший год по данным "календариков" - 27-50 дней, в среднем 30-35 дней, последние 3 цикла до вступления в исследование - без приема контрацептивных препаратов

• регулярный режим сна-бодрствования с отходом ко сну между 22:00-1:00 и пробуждением между 6:00-9:00

• относительно здоровые, без серьезных эндокринных и др. расстройств; при подозрении на гиперандрогению необходимо предоставить нормальные результаты анализов на тестостерон и 17-ОКС

• предпочтение отдавать лицам с зимне-летними колебаниями самочувствия и настроения

• согласие на участие в исследовании и возможность соблюсти все требования протокола

Критерии исключения:

• отсутствие контактного телефона

• донорство

• пересечение >2-часовых временных поясов, сменный /ночной труд и острое тяжелое заболевание - в течении последнего месяца

• прием медикаментов, которые могут влиять на гормональную систему, - последние 1-8 недель (в зависимости от медикамента).

**Схема исследования**

15

22

29

- забор крови в 15-18 ч. (и затем сбор мочи в течение 12 ч.)


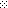


8


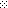


Цикл 0

Цикл 1

Цикл 2

Цикл 3

День начала месячных

0

- светолечение неярким светом, дома

в случайном

порядке

Дней цикла

- светолечение ярким светом, дома

- УЗИ

- ежедневное измерение ректальной температуры

*Цикл 0.* Отбор пациента на основании вышеуказанных критериев. Подписание Информированного согласия, которое вместе с Памяткой выдается на руки пациенту.

*Цикл 1.* День 6-9 от начала месячных. Исследуемый прибывает в медицинский центр в назначенный день в 15-18 часов дня, натошак, сдает 5 мл венозной крови, собранную накануне полусуточную мочу, проходит УЗИ яичников для оценки фолликулогенеза и забирает домой светильник и Дневник /**Приложение 5**/. Дома исследуемый проводит недельный курс светолечения, измеряет ректальную температуру, а данные заносит в Дневник. Ровно через неделю исследумый повторно сдает кровь и проходит УЗИ. Третий визит - еще через неделю, только для УЗИ. Однако, визит может пройти раньше, если по температуре появились признаки овуляции, тогда пациент звонит врачу и согласовывает дату досрочного визита.

*Цикл 2.* Свободный от исследования.

*Цикл 3.* Соблюдается точно такое же расписание, как во время цикла 1. Различие только в яркости света во время светолечения.

**Методы**

**Определение гормонов.** Кровь отстаивается 20-60 мин., центрифугируется на скорости 3000 об/мин., 2 мл сывортки собирается в эппендорф и замораживается. Пример маркировки образцов: "7d", где 7 - номер исследуемого по очереди вступления в эксперимент (в данном случае - седьмой), d - 4-й забор крови (по счету, всего - 4). Определение гормонов - ЛГ, ФСГ, эстрадиола, ТТГ и пролактина, - проводится стандартными методами после сбора всех образцов сыворотки и мочи от исследуемого.

**УЗИ** проводится врачом-гинекологом Самойловой Е., на стандартных приборах, интравагинально. Оценивается размер фолликулов, общее состояние эндометрия, наличие жидкости забрюшинно.

**Светолечение** проводится на дому в течение 7 дней подряд. Воздействие ярким светом осуществляется с помощью светильника фирмы Outside In, Англия, модель Sunray Max, который на расстоянии 41 см создает яркий свет 4600 люкс. Размер светильника - 27 x 18 x 15 см, вес 5 кг. Содержит 3 люменисцентные лампы 36 Вт с зеркальным вогнутым отражателем внутри и рассеивающим экраном снаружи. Светильник располагается на столе на расстоянии 41 см от экрана до глаз. Смотреть на экран необязательно, можно читать, вязать и т.п., но, главное, чтобы оба глаза были доступны свету. Продолжительность сеанса - 45 минут, после пробуждения, начало между 6 и 9 часами утра. В качестве контроля в другой цикл используется неяркий свет от отечественного бытового светильника, содержащего 1 люминисцентную лампу 36 Вт (интенсивность света на расстоянии 41 см - 100 люкс). Правила пользования - те же, что и при ярком свете. Побочные действия при светолечении известны, это - головная боль, раздражение глаз, - которые встречаются менее, чем в 2% случаев, и быстро нивелируются при отодвигании светильника на более дальнее расстояние или уменьшении времени экспозиции.

**Ректальная температура** измеряется исследуемым с первого же дня светолечения и продолжается до 1-го дня следующего цикла. Измерение проводится в течение 5-6 минут после пробуждения, не вставая с постели, при этом время пробуждения должно быть стабильным (± 1 час), так как температура зависит как от положения тела, так и от времени измерения.

**Анализ полученных данных**

Случаи, в результате которых пациент выводится из исследования:

• появление критериев исключения (см. выше)

• несоблюдение времени начала светолечения между 6 и 9 часами утра

• слишком короткие сеансы светолечения - < 35 минут в среднем

• недостаточное количество сеансов светолечения - < 6 за неделю

• отсутствие сыворотки и мочи для анализа в любой из 4-х *запланированных* дней забора крови

• отсутствие УЗИ данных о размере фолликула до и сразу после курса светолечения

Дисперсионный анализ rmANOVA сравнит динамику каждого из гормонов и размера фолликула между двумя циклами в результате светолечения. При p<0.05 дальнейшее сравнение между "точками" будет проводится при помощи парного *t*-теста Стьюдента. Оценка частоты овуляторных циклов проводится критерием X^2^. Для оценки связи между показателями используется метод линейной корреляции Пирсона. Данные о побочных действий светолечения собираются во время и после проведения курса светолечения и анализируются.

**III. ДОПОЛНИТЕЛЬНЫЕ СВЕДЕНИЯ**

**Календарный план**

Исследование планируется к проведению в холодное время года, когда уровень утреннего освещения низкий.

1) Сентябрь 2003 г.: Получение разрешения Этического комитета НИИ терапии СО РАМН на проведение исследования.

2) Сентябрь 2003 г. - апрель 2004 г.: Проведение исследования у первых 6-10 человек.

3) Май - июль 2004 г.: Статистическая обработка полученных данных, предварительная оценка и обсуждение результатов, корректировка методов.

4) Сентябрь 2004 г. - Апрель 2005 г.: Проведение исследования у следующих 20-30 человек.

5) Май - июль 2005 г.: Итоговая статистическая обработка полученных данных.

6) Август - декабрь 2005 г.: Отчет, печатная продукция.

**Принципы финансирования**

Проект на данный момент не имеет отдельного финансирования. Соглашение строится на безвозмездной и взаимовыгодной основе: пациентам важно выяснить причину нарушений, врачам - оценить влияние света. Все возникающие в ходе исследования необходимые затраты оплачиваются лично Даниленко К.В.

**Координаты исполнителей**

Даниленко Константин Васильевич - ответственный исполнитель, тел. р. 67-97-55, д. 37-80-39.

Самойлова Елена Анатольевна - врач-гинеколог, тел. д. 25-22-61.

Ответственный за исследование

снс, кмн Даниленко К.В.
