# Supplementary material for: Stimulatory Effect of Morning Bright Light on Reproductive Hormones and Ovulation: Results of a Controlled Crossover Trial
Source: PLoS Clin Trials. 2007 Feb 9;2(2):e7. doi: 10.1371/journal.pctr.0020007 (PMC1851732; doi:10.1371/journal.pctr.0020007)
Supplement: Trial Protocol (English) [file pctr.0020007.sd003.doc]

Inst of Internal Medicine SB RAMS

Novosibirsk

"_____" _____________ 2003

## DESCRIPTION OF THE STUDY "M-s3"

Influence of artificial light on ovulation and menstrual cycle in humans

I. GENERAL INFORMATION

A study "Influence of artificial light on ovulation and menstrual cycle in humans" is planned to be conducted by senior scientist of the Inst of Internal Medicine SB RAMS Danilenko Konstantin Vasilievich (principal investigator) and physician-gynecologist of medical centre "Tet-a-tet" Samoilova Elena Anatolievna for a period from September 2003 until May 2005. 25-40 women with lengthened menstrual cycles are invited to take part.

From 1967 to 2002, there were 8 studies performed in San-Diego and Novosibirsk on the effect of light on ovulation and menstrual cycle in women with long menstrual cycles (summarized in Putilov AA, Danilenko KV, Protopopova AY, Kripke DF. Menstrual Phase Response to Nocturnal Light. Biol Rhythm Res, 2002, 33:23-38). Since 1989, the shortening of menstrual cycle in response to light therapy has been consistently tracked in patients with winter depression (Danilenko, unpublished data). Nevertheless, ultrasound and hormonal investigation, as the most objective methods of verification of obtained results, were not used in any of these studies. In 2003, preliminary data were received that light stimulates excretion of luteinizing hormone in men (Yoon et al., 2003).

**Aim:** To investigate an influence of artificial bright light on ovulation and menstrual cycle in humans.

**Tasks:**

(1) to investigate the influence of bright light on secretion of several hormones

(2) to investigate the influence of bright light on follicle development, ovulation and menstrual cycle length.

### II. SUBJECTS AND METHODS

#### Subjects

25-40 women with lengthened menstrual cycles are planned to take part. Their search is via advertisements in local newspapers /**Enclosure 1**/ and a flow of gynecologic patients in the profiled medical settings of the city. After completing a Questionnaire /**Enclosure 2**/ and reading the Study Outline /**Enclosure 3**/, volunteers are selected during a face-to-face interview with principal investigator Danilenko K.V. and doctor Samoilova E.A. The Consent form is signed /**Enclosure 4**/ if the candidate suits all inclusion and exclusion criteria.

Inclusion criteria:

• age 18-40 years

• body mass index 18-30 kg/m^2^

• menstrual cycle length from the diaries is 27-50 days, on average 30-35 days, the last 3 cycles before entering the study are free from contraceptive medications

• a regular regimen of sleep-wakefulness with sleep onset between 22:00-1:00 and awakening between 6:00-9:00

• relatively healthy, without serious endocrine and other disorders; if there is a suspicion for hyperandrogeny, normal results of testosterone and 17-hydroxyprogesterone should be provided

• persons with winter-summer fluctuations in self-feeling and mood are in favor

• a willingness to participate in the study and adhere to the requirements of the protocol

Exclusion criteria:

• absence of contact telephone

• blood donation

• crossing >two time zones, shift/night work or acute severe illness during the last month

• taking medication known to interfere with hormonal system during the last 1-8 weeks (depending on medication).

**A scheme of the study:**


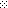

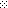


15

22

29

- blood sample at 15-18 h. (followed by urine collection during 12 h)

8

Cycle 0

Cycle 1

Cycle 2

Cycle 3

a day of menstruation onset

0

- light exposure with dim light, at home

in random

order

Days of cycle

- light exposure with bright light, at home

- USI

- daily recording of rectal temperature

*Cycle 0*: Inclusion of the participant based on the above criteria. Signing the Consent form which is given to participant at home together with the Study Outline.

*Cycle 1*: The 6th-9th day of the cycle. The participant attends the medical centre on the arranged day at 15:00-18:00, gives 5 ml of fasted venous blood, brings a half-day overnight urine collection,

undergoes an USI (ultrasound investigation) to assess follicle growth, and takes a light device and a Diary /**Enclosure 5**/ back home. At home, participant receives a week’s course of light exposure, measures rectal temperature, and records the data in the Diary. After exactly one week, the participant again provides a blood sample and undergoes USI. The participant’s third visit is after a further week and involves USI only. However, the participant should arrange to attend earlier if rectal temperature readings indicate that ovulation may have occurred.

*Cycle 2*: No investigation.

*Cycle 3*: Exactly the same schedule as Cycle 1. The only difference is the brightness of light during light exposure.

## Methods

**Hormonal assay.** The blood is left to settle for 20-60 min, centrifuged at 3000 revolutions per minute, 2 ml of serum is collected in an Eppendorf tube and frozen. An example of labelling: ‘7d’, where ‘7’ is the participant's unique reference number and a, b, c or d relates to each of their four blood samples. The hormonal assay for LH, FSH, estradiol, TSH and prolactin is performed using standard methods when all four blood and urine samples are collected from the participant.

Transvaginal **USI** is carried out by physician-gynecologist Samoilova E. using standard apparatus. Follicle size, endometrium thickness and presence of free fluid behind the uteri were determined.

**Light exposure** takes place at home for 7 consecutive days. The intervention with bright light is carried out by means of light device from Outside In (England), model Sunray Max, that provides a light intensity of 4600 lux at a distance of 41 cm. The size of the light device is 27 x 18 x 15 cm, weight is 5 kg. It contains three fluorescent 36 W bulbs with a glass-mirrored concave reflector behind them - inside, and a diffusing screen - outside. The light device is placed on a table at a distance of 41 cm from the screen to the eyes. It is not necessary to look at the screen all the time - one is able to read, knit etc. but it is important that both eyes remain open to light. Light exposure starts between 06:00 – 09:00, shortly after waking and each session takes 45 minutes. The non-bright light control in the other study cycle used a domestic fluorescent fitting containing one 36 W tube (light intensity at a distance of 41 cm is 100 lux). Guidelines for use were similar to those for bright light exposure. Adverse events during light therapy (e.g. headache, eyestrain) are well known and relatively minor. Their occurrence rate is less than 2% and they usually disappear simply by positioning the light device slightly further away or by reducing the exposure time.

**Rectal temperature** is recorded by participants from the first day of light exposure and continued until the 1st day of the next cycle. A reading is taken for 5-6 minutes after wake up, whilst still lying in bed and at the same time each day (± 1 hour) since the temperature depends both on body position and the time of the measurement.

### Analysis of the data

Reasons for discontinuing participation:

• appearance of exclusion criteria (see above)

• not adhering to the light exposure start time between 06:00-09:00

• reduced light exposure time - <35 min on average

• insufficient number of light exposure sessions - <6 for a week

• no serum for analysis in any of 4 *planned* days of blood drawing

• no USI data on follicle size before and just after a course of light exposure

Analysis of variance mANOVA will compare the dynamics of each hormone and follicle size between two cycles of light exposure. When p<0.05, the further comparison between "points" will be performed using Student's paired *t*-test. The number of ovulation will be compared using X^2^ criterion. A link between outcomes will be tested using Pearson linear correlation analysis. Adverse events from light exposure are tracked during and after the course of light exposure, and analyzed.

**III. ANCILLARY INFORMATION**

## Calendar plan

The study is planned for a dark season when the level of morning light is low.

1) September 2003: Obtaining an approval from the Ethics Committee of the Institute of Internal Medicine SB RAMS to perform the study.

2) September 2003 - April 2004: Investigation of the first 6-10 participants.

3) May - July 2004: Statistical analysis of the data, preliminary estimation and discussion of the results, correction of the protocol.

4) September 2004 - April 2005: Investigation of the next 20-30 participants.

5) May - July 2005: Final statistical analysis of the data.

6) August - December 2005: Study report, publication.

## Principles of funding

At the moment, the project has no specific financial support. Participation is on a mutually beneficial basis: the patients receive information regarding their health problems, whilst the clinicians are able to investigate the effects of light. All necessary expenses at the study are covered by Danilenko K.V.

##### Investigator details

Danilenko Konstantin Vasilievich - principal investigator, tel. w. 67-97-55, h. 37-80-39.

Samoilova Elena Anatolievna - physician-gynecologist, tel. h. 25-22-61.

Principal investigator

senior scientist, MD, Danilenko K.V. */signature/*
